# Prevalence of Arsenic Exposure from Drinking Water and Awareness of Its Health Risks in a Bangladeshi Population: Results from a Large Population-Based Study

**DOI:** 10.1289/ehp.7903

**Published:** 2005-10-26

**Authors:** Faruque Parvez, Yu Chen, Maria Argos, A.Z.M. Iftikhar Hussain, Hassina Momotaj, Ratan Dhar, Alexander van Geen, Joseph H. Graziano, Habibul Ahsan

**Affiliations:** 1 Mailman School of Public Health, Columbia University, New York, New York, USA; 2 National Institute of Preventive and Social Medicine, Dhaka, Bangladesh; 3 Department of Chemistry, Queens College, New York, New York, USA; 4 Lamont Doherty Earth Observatory, Columbia University, Palisades, New York, USA

**Keywords:** arsenic, awareness, Bangladesh, drinking water, environmental health, public health

## Abstract

We conducted a population-based prevalence survey in Araihazar, Bangladesh, to describe the distribution of arsenic exposure in a rural Bangladeshi population and to assess the population’s awareness to this problem as well as to possible remediation options. Water samples from 5,967 contiguous tube wells in a defined geographic area were tested using laboratory-based methods. Additionally, for each well, the owner/caretaker (or a close relative) was interviewed regarding his or her awareness of the health consequences of As exposure. Arsenic exposure data and demographic characteristics for the 65,876 users of these wells were also collected from the 5,967 respondents. Among the 65,876 residents, more than half (54%) regularly consumed well water with an As concentration ≥ 50 μg/L—above the acceptable government standard in Bangladesh. Respondents were 15–92 years of age, with an average age of 42 years, and 43% were male. Presence of awareness was significantly related to male sex, nonlabor head of household occupation, better housing, and having had the well tested for As concentration. Most respondents (92%) expressed a willingness to take steps to reduce their exposure, with switching to a safe well the most favored option (46.2%). Willingness to reduce exposure was positively related to awareness of the health risks of As. However, the association between awareness and switching to a safe well [odds ratio (OR) = 1.25; 95% confidence interval (CI), 1.01–1.54] was no stronger than the associations between awareness and using surface water (with or without treatments) (OR = 1.54; 95% CI, 1.22–1.95) or using an existing well after treatment or increasing the depth (OR = 1.34; 95% CI, 1.08–1.67). These findings suggest that health education programs may need to target individuals with lower socioeconomic status and that well switching should be encouraged with more appropriate health education. Increasing knowledge of the health consequences of As may be an important element in facilitating remediation.

Chronic arsenic exposure is associated with many human health conditions, including skin lesions and cancers of the liver, lung, bladder, and skin (Ahsan et al. 2000; Guha Mazumder et al. 1998; Haque et al. 2003; Smith et al. 1998), as well as other noncancer health effects, such as adverse reproductive outcomes, neurologic disorders, and impaired cognitive development in children (Ahmad et al. 2001; Calderon et al. 2001; Mukherjee et al. 2003; Wasserman et al. 2004). Inorganic As is a natural element of the earth’s crust. More than 100 million people worldwide have been estimated to be chronically exposed to As from drinking water containing high As levels (Alaerts et al. 2001; Chowdhury et al. 2000; Dhaka Community Hospital Trust 2005). Although > 20 countries have been affected by As contamination of drinking water, the situation is perhaps the most devastating in Bangladesh because of the number of affected people. Among the country’s 7–11 million hand-pumped tube wells, approximately half have been estimated to supply groundwater with an As concentration > 50 μg/L—the maximum allowable limit in drinking water in Bangladesh (Bangladesh Arsenic Mitigation Water Supply Project 2006; Josephson 2002). Among the country’s total population of 130 million, 35 million people are believed to be exposed to an As concentration in drinking water > 50 μg/L, and 57 million people to a concentration > 10 μg/L, and thus are at higher risk of developing cancer and other As-related, life-threatening conditions [British Geological Survey (BGS) 2001; Dhaka Community Hospital Trust 2005; Hoque et al. 2000; Milton and Rahman 1999].

Although the exact time of onset of As exposure in Bangladesh is unknown, it is suspected to have started during the 1960s and 1970s when the United Nations Children’s Fund (UNICEF), in collaboration with the Bangladeshi government, started to install hand-pumped tube wells to provide pathogen-free drinking water to the population (Smith et al. 2000). Unsafe levels of As in ground-water were first detected in 1983 in Bangladesh; subsequently, however, only a small proportion of the country’s tube wells have been tested for As (Dhaka Community Hospital Trust 2005). There is wide geographic variability in the As concentration of groundwater. The percentage of As-contaminated wells ranges from as low as 0% to as high as 99% of wells, depending on the region (BGS 2001; Chowdhury et al. 2000; Dhaka Community Hospital Trust 2005; Kurokawa et al. 2001). The wide variation in As concentrations, even within a small geographic area, has made it very difficult to assess the exact magnitude of the As problem in terms of number and severity of affected people.

Until recently, little systematic research has been undertaken to assess the prevalence of As exposure or awareness of the problem in the population. Surveys conducted within highly contaminated areas have found that 17–35% of the population examined have skin lesions, and up to 3.4% of them have gangrene and ulcers (Hassan et al. 2005). The World Health Organization (WHO) has estimated that at least 5 million individuals will have As-induced skin lesions in Bangladesh within the next 5–10 years (WHO 2001a). A recent study from our group (Chen and Ahsan 2004) predicted that the future burden of deaths from internal cancers would at least double in Bangladesh because of As exposure from drinking water.

Arsenic contamination has had a profound impact at both the individual and community levels. Reports have attributed disease and death caused by As toxicity to lack of knowledge about the source of this metal (Hadi 2003). Fear of contagiousness has separated families, created social isolation in schools, and led to avoidance of people living in highly contaminated regions (Dhaka Community Hospital Trust 2005). Therefore, it is important to understand people’s perception and awareness of the adverse effects of As in order to tackle the psychosocial and health impacts and to ultimately reduce disease burden and avoidable deaths. Awareness is also important for strategies that could potentially reduce exposure. Without appropriate knowledge of the adverse health effects of As exposure and mitigation options, people will not be motivated to participate in interventions initiated by governmental and nongovernmental agencies.

As a first step in establishing the Health Effects of Arsenic Longitudinal Study (HEALS), a large prospective cohort study, we undertook a population-based survey where all 5,967 contiguous wells in a geographically defined area of Bangladesh were tested and their owners/users interviewed. In this article, we present the findings of this comprehensive survey, with a particular focus on the prevalence and awareness of As exposure as well as factors associated with exposure awareness in a rural Bangladeshi population.

## Materials and Methods

We conducted a population-based survey between March and June 2000, in three unions of Araihazar Upazila, Bangladesh—an area 25 km east of the capital city, Dhaka. The goal of this survey was to completely enumerate and characterize the tube wells and their users in the study area to create a sampling frame for recruiting participants into the HEALS cohort. From a 25-km^2^ area in Araihazar, water samples were collected from 5,967 contiguous tube wells, and their owners/caretakers were interviewed to obtain demographic characteristics on the 65,876 users of these wells. We selected this study area because of the wide variation in well-water As concentrations. A detailed description of the HEALS methodologies, including selection of the study area and population, is reported elsewhere (Ahsan et al. 2005).

For this population-based survey, six teams of trained male and female interviewers and well-water samplers went to every *bari* in the defined study area. A *bari* refers to a cluster of households that reside closely. Many individual households do not possess a tube well, but at least one well was present in each *bari* in this study area. After the identification of each well and its owner/caretaker within a *bari*, the field team performed the two major components of the survey. The first component was to collect water samples and geographic positioning system data for each well. Water samples were collected in acid-washed polyethylene bottles and were transported to Columbia University, where total As was measured by graphite-furnace atomic absorption spectrometry with a detection limit of 5 μg/L. A detailed description of the water sampling, processing, quality control, chemicals used, and analyses has been published elsewhere (Van Geen et al. 2002).

The second component was an in-person interview with the well owner/caretaker (or a close relative, if the owner/caretaker was not available) using a structured questionnaire. Sociodemographic characteristics, occupation of the head of the household, and respondent’s awareness of and possible solutions for the As problem were ascertained from one respondent (well owner/caretaker or close relative) for each well. Eighty-eight percent of respondents were the well owner/caretaker, and 12% were other close relatives living in the same household with the well owner/caretaker. Occupation of the head of the household was defined as the job where the person spent the most time working in the past year, indicative of the main source of household income for the past year. Although 57% of the interviewees were female, the head of the household was usually male.

Knowledge regarding the health risks of As was assessed by asking whether the respondent was aware of any adverse health effects from As in drinking water. Specifically, the respondents were asked the following question: “Are you aware that drinking As-contaminated water may cause adverse health effects?” The answers were recorded as “yes,” “no,” or “don’t know.” Answers of “no” and “don’t know” were combined into a single category for the purposes of this analysis. Those who answered “yes” were asked to further specify As-related diseases or adverse health effects. This was an open-ended question for which responses were subsequently categorized by the study physicians to simplify the analysis.

Study participants were also asked about options they were willing to take if As was found in their well. There were 11 mutually exclusive choices listed in the questionnaire: *a*) will not do anything, *b*) use dug-well water, *c*) use pond water, *d*) boil well/pond water, *e*) use rain water, *f* ) boil tube-well water, *g*) settle tube-well water, *h*) increase the depth of the well, *i*) use filter, *j*) switch well, and *k*) unknown. Five categories were created based on these 11 choices: do nothing, use surface water with or without treatments (combined *b*–*e* ), use existing well after treatment or increasing the depth (combined *f*–*i*), switch to safe well, and unknown.

In addition, the well owners/caretakers or their close relatives were asked for information on the number of regular users of the tube well, as well as demographic and family characteristics of the users, in order to assess the As exposure distribution among the overall population in the study area.

### Statistical analysis.

Descriptive analyses involved calculations of frequency distributions, means/medians, and tabular statistics. Categories were created for age. All other variables for demographic characteristics and factors related to well use were categorical. We compared distributions of demographic characteristics and factors related to well use between subjects with and without exposure awareness using chi-square tests. A major goal of this analysis was to determine the prevalence of awareness of the health risks of As and to determine factors that are associated with this awareness. We estimated adjusted odds ratios (ORs) and their 95% confidence intervals (CIs) to assess the strength of the associations of awareness of As problems (dependent variable) with different sociodemographic and mitigation-related variables (independent variables) using unconditional logistic regression models. Study participants with missing information on any of the covariates were excluded from logistic regression analysis (*n* = 825). All statistical analyses were conducted using SAS software (version 8.2; SAS Institute Inc., Cary, NC, USA).

## Results

The demographic characteristics of the study participants are shown in Table 1. Among the 5,967 tube-well owners/caretakers or their close relatives interviewed, 57% were female and 43% were male. The average age of respondents was 42 years. A large percentage of respondents owned or were employed in small businesses (40%); others worked in textile factories (15.6%), other paid employment (18%), and agriculture (25%). Only one tube well was typically present in each *bari* (72%), with only 28% having more than one (Table 1). The average number of tube wells was 1.9 per *bari*. More than half (61%) of the well owners/caretakers or their close relatives reported they were aware of health problems associated with As from drinking water. Nearly 89% of those aware of the problem (2,891 of 3,631) associated As with skin disorders (Table 1).

The distribution of As exposure among the 65,876 residents in the study area is presented in Table 2. Approximately 53% of residents in the study area drank water with an As concentration > 50 μg/L, and nearly 75% of the population drank water with a concentration > 10 μg/L (Table 2).

The adjusted ORs for the association between awareness of health consequences and several sociodemographic variables are shown in Table 3. These variables include age, sex, occupation of the head of household, socioeconomic status (SES), status of tube-well testing, and mitigation option preferences. Age of the respondents was associated with awareness, with the highest awareness observed among those 30–44 years of age (OR = 1.21; 95% CI, 1.02–1.43) and the lowest among those ≥ 60 years of age (OR = 0.70; 95% CI, 0.56–0.88). Male respondents were more likely to be aware of health risks than female respondents (OR = 1.29; 95% CI, 1.14–1.47).

We observed strong associations between the respondents’ awareness and measures of their SES, including occupation of the head of the household and house type. Those with higher income or occupations related to higher education were more likely to be aware of the health problems of As. Compared with agricultural laborers (considered the lowest socioeconomic group by occupation in rural Bangladesh), respondents who were paid employees (OR = 3.66; 95% CI, 2.38–5.65), small business owners/employees (OR = 3.39; 95% CI, 2.23–5.14), farmers with agricultural land (OR = 2.26; 95% CI, 1.47–3.47), daily contract laborers (OR = 1.90; 95% CI, 1.21–2.99), or factory workers (OR = 1.89; 95% CI, 1.23–2.90) were more likely to be aware of As-related health problems. Similarly, better living conditions were positively associated with the awareness of health risks. Compared with those living in thatched houses (houses with the poorest living condition in the area), respondents living in brick (OR = 4.37; 95% CI, 3.14–6.07), partial brick (OR = 4.27; 95% CI, 3.18–5.74), corrugated tin (OR = 2.16; 95% CI, 1.73–2.69), or other nonthatched houses (OR = 2.00; 95% CI, 1.39–2.88) were more likely to be aware of As-related health problems. The number of wells in the *bari* was not associated with respondent awareness.

Willingness to adopt any mitigation options and/or to have the well tested for As was associated with awareness of As-related health problems. Those who had previously tested their wells for As were nearly three times (OR = 2.70; 95% CI, 2.05–3.57) more likely to be aware of the health effects of As compared with those who had not tested their wells. Similarly, respondents willing to adopt one of the major mitigation options were more likely to be aware of the health effects of As (ORs ranging between 1.25 and 1.54) compared with those who preferred to do nothing. However, the association between awareness and switching to a safe well (OR = 1.25; 95% CI, 1.01–1.54) was no stronger than the associations between awareness and using surface water (with or without treatments) (OR = 1.54; 95% CI, 1.22–1.95) or using existing well after treatment or increasing the depth (OR = 1.34; 95% CI, 1.08–1.67).

## Discussion

In the present study, we found that 35 and 54% of the 65,876 residents in the study were exposed to drinking water with As concentrations exceeding 100 μg/L and 50 μg/L (the Bangladesh government standard), respectively. Almost three-fourths had been drinking water with an As concentration above the WHO recommended level of 10 μg/L (WHO 2001b). We interviewed 5,967 well owners/caretakers or their close relatives and found that awareness of the health effects of As exposure was independently associated with factors including age, sex, occupation of household head, type of house, and prior testing of tube well for As concentration. In addition, awareness of health effects of As was positively associated with remediation options, including the use of surface water, treatment of contaminated well/well water, and well switching, compared with doing nothing.

The extent of As exposure in this study area is comparable with what has been reported in other national surveys, although previous surveys did not assess the exposure distribution in a systematic manner to cover every well and associated users within a given area. The percentage of wells with an As concentration > 50 μg/L in other studies ranged from 30% (BGS 2001) to 59% (Chowdhury et al. 2000); the percentage > 300 μg/L ranged from 8.4% (BGS 1998) to 20% (Chowdhury et al. 2000). In our study area, the percentages of the wells with an As concentration > 50 μg/L and > 300 μg/L were 54.5% and 6.8%, respectively. Field kits have been used in many of the previous surveys to test for well-water As concentration. The lack of reliability and sensitivity of these kits complicates exposure assessment. Most of the field kits are not able to quantitate As concentrations < 100 μg/L. Additionally, one recent report showed 45% of 2,866 wells tested by both field kits and standard laboratory methods were found to be discrepantly labeled either as safe or unsafe (Rahman et al. 2002).

We found that 61% of study participants were aware of at least some of the health effects of As. This estimate is lower than what has been seen in two very small previous studies. Rahman (2002) found that 82% of respondents (*n* = 224) in rural Bangladesh associated As exposure with adverse health effects, and Hanchett et al. (2002) reported that 75% of all the female respondents (*n* = 97) were aware of the As problem. In both studies, however, surveys were carried out after water samples were tested and patients with skin lesions in the study area were identified. In the present study, the study area had not received much information locally regarding the As problem compared with the other regions in Bangladesh that have been surveyed previously. Various health education campaigns were implemented after the well sampling and testing by the HEALS because of ethical obligations (Ahsan et al. 2005). Participants were informed of the As concentration in their well only after all the sampling and testing were finished. A four-member team disseminated a culturally appropriate and acceptable message regarding As-related health information to the entire study area at the community level over a 5-month period. Because these measures were taken after the completion of the household survey and well sampling, it is unlikely that they have biased our study results.

Eighty-nine percent of subjects who were aware of potential health effects of As exposure associated As exposure with skin lesions. A recent study by a large nongovernmental organization found that knowledge about the As problem was related to a prior experience of seeing an afflicted patient (BRAC 2000). We did not assess the health conditions of well owners/care takers in the present study. Because definition of arsenicosis varies, we did not ask respondents whether they knew an individual affected by As toxicity. Therefore, we were unable to evaluate whether awareness was related to participant’s skin lesion status or knowledge of a patient with arsenicosis. In the large cohort study, HEALS—which was initiated using the sampling frame based on this present study—the health conditions of its study participants were assessed (Ahsan et al. 2005). Skin lesions are the most common manifestation of As toxicity (Alain et al. 1993; Yeh 1973), and this was also found in the HEALS cohort (Ahsan et al., in press). Epidemiologic literature has shown As to be associated with a wide array of health conditions, including cancers and cardiovascular and neurologic diseases. Health education programs should also provide information on other potential health outcomes due to As exposure in order to enhance awareness and encourage behavioral changes such as well switching.

Our study clearly indicates that people with higher SES (nonlabor occupation of the head of the household and better housing) were more aware of the health effects of As. This is consistent with the findings of Hadi (2003), who reported SES variables that were related to the knowledge of the health problems of As exposure. A previous study (Hadi and Parveen 2004) and research from our group (Argos M, Parvez F, Chen Y, Hussain AZMI, Momotaj H, Howe GR, Graziano JH, Ahsan H, unpublished data) have found that patients with As-related skin lesions were more likely to have a lower SES. Taken together, health education programs should consider targeting individuals with lower SES or be designed with the characteristics of this subpopulation in mind. In the present study, we also found that males were more aware of As-related health problems than females, which is also consistent with the findings of Hadi (2003) and Hanchett et al. (2002). This may possibly be due to the higher disease burden in males or other cultural differences between men and women.

Research of other health issues has shown that awareness is related to knowledge of a correct behavioral or lifestyle modification (Eloundou-Enyegue et al. 2005; Piechulek et al. 2003). Previous studies have not associated awareness with preference of well switching in comparison with other remediation actions (Hadi 2003; Hanchett et al. 2002). In the present study, we observed that knowledge of a correct remediation option, switching to a safe well, was insufficient. For example, > 25% of respondents reported boiling water as a strategy to avoid the health risks from As, likely due to the past promotion of boiling water to reduce the prevalence of diarrhea and other waterborne diseases (Hanchett et al. 2002). In addition, although awareness was positively related to each of the remediation options compared with doing nothing, we also found that the positive association between well switching and awareness was no stronger than the associations between other erroneous mitigation options (using surface water or using an existing well with or without treatment) and awareness. Individuals reporting awareness of the health consequences of As were willing to adopt mitigation actions; however, it appeared that individuals with awareness were not able to all choose a correct remediation option, possibly due to lack of health education specifically addressing As mitigation. These findings suggest that the reinforcement and implementation of health education and health policy are still needed in a population with moderate awareness because misconceptions still persist.

The strengths of this study include rigorous study methodology, comprehensiveness of well coverage, and laboratory-based As concentration analyses. The work was conducted in an area where little prior As work had taken place. Because people were interviewed before they knew the As concentration of their well, the survey results—regarding exposure (number of people regularly drinking from the wells) and the extent of knowledge about the As problem—are likely to be unbiased.

Despite the many strengths of this study, there are some potential limitations. First, the survey was conducted in a relatively small geographic area (25 km^2^) with a population of about 65,876. We found that the extent of As exposure in the study was comparable with estimates from previous surveys using accurate laboratory-based methods (graphite-furnace atomic absorption spectrometry). Precise estimates of the distribution of As for the whole nation have yet to be determined. Such an undertaking would require the participation of local governments at the village level. Second, in this study we interviewed either a tube-well owner or caretaker or a close relative. The study population (well owners and caretakers) would be expected to have a higher SES on average compared with the general population of Bangladesh. Thus, the percentage of awareness in the general population would be expected to be lower. Last, we acknowledge that an in-depth interview would reveal more information about awareness of the As problem and selection of remediation options in this population. For instance, information on levels of awareness and reasons for selecting different remediation options (particularly financial and social) would be valuable for planning health education programs. In the near future, we plan to assess these research questions in the HEALS.

In summary, using laboratory-based As concentration analyses and detailed household surveys, we found a large extent of high As exposure in the study area. Awareness of As-related health effects was positively related to male sex, better housing, and nonlabor occupation of the head of household among the 5,967 well owners/caretakers or their close relatives. Awareness is positively related to remediation options compared with doing nothing. Educating the population about As-related health problems is a necessary and important first step in abating this problem. Increasing public awareness and then offering practical mitigation options are excellent ways to begin making inroads in fighting this massive public health problem.

## Figures and Tables

**Table 1:** Distribution of respondents’ sociodemographic characteristics and other key variables, by awareness of health consequences from drinking As-contaminated water.

		Aware of health effects of As		
Characteristic	Total participants^a^*n* (column %)	Yes^b^*n* (row %)	No/don’t know^c^*n* (row %)	*p*-Value^d^
Age (years)
15–29	830 (15.2)	483 (58.2)	347 (41.8)	< 0.01
30–44	2,269 (41.7)	1,448 (63.8)	821 (36.2)	
45–59	1,613 (29.6)	960 (59.5)	653 (40.5)	
60–90	733 (13.5)	398 (54.3)	335 (45.7)	
Unknown	522	342	180	
Sex
Female	3,390 (56.8)	2,019 (59.6)	1,371 (40.4)	0.02
Male	2,577 (43.2)	1,612 (62.6)	965 (37.4)	
Occupation of the head of the household
Agricultural labor	112 (1.7)	38 (33.9)	74 (66.1)	< 0.01
Daily contract laborer	425 (7.2)	209 (49.2)	216 (50.8)	
Factory worker	935 (15.8)	458 (49.0)	477 (51.0)	
Farmer (with own land)	970 (16.4)	524 (54.0)	446 (46.0)	
Small business owner/employee	2,392 (40.3)	1,609 (67.3)	783 (32.7)	
Other paid jobs	1,093 (18.4)	766 (70.1)	327 (29.9)	
Unknown	40	27	13	
Type of house
Thatched	401 (6.8)	151 (37.7)	250 (62.3)	< 0.01
Corrugated tin	4,196 (70.6)	2,475 (59.0)	1,721 (41.0)	
Semi *pakka* (partial brick)	641 (10.8)	495 (77.2)	146 (22.8)	
*Pakka* (brick)	498 (8.4)	377 (75.7)	121 (24.3)	
Other nonthatched types	203 (3.4)	112 (55.2)	91 (44.8)	
Unknown	28	21	7	
No. of tube wells in the *bari*
1	4,312 (72.3)	2,554 (59.2)	1,758 (40.8)	< 0.01
> 1	1,655 (27.7)	1,077 (65.1)	578 (34.9)	
Tube-well water previously tested for As
No	5,560 (93.2)	3,300 (59.4)	2,260 (40.6)	< 0.01
Yes	407 (6.8)	331 (81.3)	76 (18.7)	
Steps to be taken if As found in tube-well water
Do nothing	181 (3.2)	77 (42.5)	104 (57.5)	< 0.01
Use surface water with or without treatments	1,140 (20.2)	746 (65.4)	394 (34.6)	
Use existing well after treatment or increasing the depth	1,716 (30.4)	1,062 (61.9)	654 (38.1)	
Switch wells	2,610 (46.2)	1,547 (59.3)	1,063 (40.7)	
Unknown	320	199	121	
Specific health conditions mentioned by participants who were aware of health risks (column %)
Skin changes		2,891 (88.8)		
Neurologic disorders		23 (0.7)		
General bad health		253 (7.7)		
Other specific diseases		90 (2.8)		
Unknown		374		

a *n* = 5,967 total participants.

b *n* = 3,631 “yes” responses.

c *n* = 2,336 “no/don’t know” responses.

d *p*-Value for chi-square test comparing participants who were aware of health effects of As with those who were not. Unknowns were not included in statistical comparison.

**Table 2:** Arsenic exposure distribution among residents of Araihazar, Bangladesh.

As concentration (μg/L)	People regularly using the tube wells for drinking/cooking *n* (%)	Wells/respondents^a^*n* (%)
5–9	17,271 (26.2)	1,476 (24.7)
10–49	13,052 (19.8)	1,240 (20.8)
50–99	11,960 (18.2)	1,132 (19.0)
100–199	12,397 (18.8)	1,158 (19.4)
200–399	9,841 (14.9)	828 (13.9)
400–864	1,355 (2.1)	132 (2.2)
Total	65,876 (100.0)	5,966 (100.0)

a One well had unknown As concentration and was excluded from the analysis.

**Table 3:** Adjusted ORs for awareness of health consequences from drinking As-contaminated water in relation to sociodemographic characteristics.

Characteristic	No. aware/not aware (total *n* = 5,142)	Adjusted ORs for awareness (95% CI)
Age (years)^a^
15–29	453/337	1.00
30–44	1,365/778	1.21 (1.02–1.43)
45–59	912/613	0.96 (0.80–1.16)
60–90	374/310	0.70 (0.56–0.88)
Sex^a^
Female	1,789/1,240	1.00
Male	1,315/798	1.29 (1.14–1.47)
Occupation of the head of the household^a^
Agricultural labor	33/70	1.00
Daily labor	189/197	1.90 (1.21–2.99)
Factory worker	401/400	1.89 (1.23–2.90)
Farmer	473/405	2.26 (1.47–3.47)
Small business owner/employee	1,396/690	3.39 (2.23–5.14)
Other paid jobs	612/276	3.66 (2.38–5.65)
Type of house^a^
Thatched	136/231	1.00
Corrugated tin	2,180/1,526	2.16 (1.73–2.69)
Semi *pakka* (partial brick)	402/117	4.27 (3.18–5.74)
*Pakka* (brick)	286/81	4.37 (3.14–6.07)
Other type	100/83	2.00 (1.39–2.88)
No. of tube wells in the *bari*^a^
1	2,167/1,516	1.00
> 1	937/522	1.06 (0.93–1.20)
Tube-well water previously tested for As^a^
No	2,828/1,975	1.00
Yes	276/63	2.70 (2.05–3.57)
Steps to be taken if As found in tube-well water^a^
Do nothing	69/97	1.00
Use surface water with or without treatments	670/363	1.54 (1.22–1.95)
Use existing well after treatment or increasing the depth	951/604	1.34 (1.08–1.67)
Switch wells	1,414/974	1.25 (1.01–1.54)

a ORs were adjusted for all other social demographic characteristics.

## References

[b1-ehp0114-000355] Ahmad SA, Sayed MH, Barua S, Khan MH, Faruquee MH, Jalil A (2001). Arsenic in drinking water and pregnancy outcomes. Environ Health Perspect.

[b2-ehp0114-000355] Ahsan H Chen Y Parvez F Argos M Hussain A Momotaj H2005. Health Effects of Arsenic Longitudinal Study (HEALS) description of a multidisciplinary epidemiologic investigation. J Expo Anal Environ Epidemiol 10.1038/sj.jea.7500449 [Online 14 September 2005]. 10.1038/sj.jea.7500449 16160703

[b3-ehp0114-000355] Ahsan H Chen Y Parvez F Zablotska L Argos M Hussain A In press. Arsenic exposure from drinking water and risk of premalignant skin lesions in Bangladesh: baseline results from the Health Effects of Arsenic Longitudinal Study (HEALS). Am J Epidemiol. 10.1093/aje/kwj154 16624965

[b4-ehp0114-000355] Ahsan H, Perrin M, Rahman A, Parvez F, Stute M, Zheng Y (2000). Associations between drinking water and urinary arsenic levels and skin lesions in Bangladesh. J Occup Environ Med.

[b5-ehp0114-000355] Alaerts G Khouri N Kabir B 2001. Strategies to mitigate arsenic contamination of water supply. In: Arsenic in Drinking Water. United Nations Synthesis Report on Arsenic in Drinking Water. Available: http://www.who.int/water_sanitation_health/dwq/arsenicun8.pdf [accessed 4 June 2005].

[b6-ehp0114-000355] Alain G, Tousignant J, Rozenfarb E (1993). Chronic arsenic toxicity. Int J Dermatol.

[b7-ehp0114-000355] Bangladesh Arsenic Mitigation Water Supply Project (BAMWASP) 2006. Available: http://www.bamwsp.org [accessed 2 February 2006].

[b8-ehp0114-000355] BGS (British Geological Survey) 1998. Groundwater Studies for Arsenic Contamination in Bangladesh—Phase 1 Findings. Available: http://www.bgs.ac.uk/arsenic/bphase1/B_find.htm [accessed 2 February 2006].

[b9-ehp0114-000355] BGS (British Geological Survey) 2001. Arsenic Contamination in Groundwater in Bangladesh—Phase 1. Vol 1: Summary (Kinniburgh DG, Smedly PL, eds). Available: http://www.bgs.ac.uk/arsenic/Bangladesh/Reports/Vol1Summary.pdf [accessed 2 February 2006].

[b10-ehp0114-000355] BRAC 2000. Combating A Deadly Menace. Early Experience with a Community-Based Arsenic Mitigation Project in Bangladesh. Research Monograph no. 16. Dhaka, Bangladesh:BRAC Research and Evaluation Division.

[b11-ehp0114-000355] Calderon J, Navarro ME, Jimenez-Capdeville ME, Santos-Diaz MA, Golden A, Rodriguez-Leyva I (2001). Exposure to arsenic and lead and neuropsychological development in Mexican children. Environ Res.

[b12-ehp0114-000355] Chen Y, Ahsan H (2004). Cancer burden from arsenic in drinking water in Bangladesh. Am J Public Health.

[b13-ehp0114-000355] Chowdhury UK, Biswas BK, Chowdhury TR, Samanta G, Mandal BK, Basu GC (2000). Groundwater arsenic contamination in Bangladesh and West Bengal, India. Environ Health Perspect.

[b14-ehp0114-000355] Dhaka Community Hospital Trust 2005. Arsenic Problem in Bangladesh. Available: http://www.dchtrust.org/arsenic_problem.htm [accessed 7 May 2005].

[b15-ehp0114-000355] Eloundou-Enyegue PM, Meekers D, Calves AE (2005). From awareness to adoption: the effect of AIDS education and condom social marketing on condom use in Tanzania (1993–1996). J Biosoc Sci.

[b16-ehp0114-000355] Guha Mazumder DN, Haque R, Ghosh N, De BK, Santra A, Chakraborty D (1998). Arsenic levels in drinking water and the prevalence of skin lesions in West Bengal, India. Int J Epidemiol.

[b17-ehp0114-000355] Hadi A (2003). Fighting arsenic at the grassroots: experience of BRAC’s community awareness initiative in Bangladesh. Health Policy Plan.

[b18-ehp0114-000355] Hadi A, Parveen R (2004). Arsenicosis in Bangladesh: prevalence and socio-economic correlates. Public Health.

[b19-ehp0114-000355] Hanchett S, Nahar Q, Van Agthoven A, Geers C, Rezvi MD (2002). Increasing awareness of arsenic in Bangladesh: lessons from a public education programme. Health Policy Plan.

[b20-ehp0114-000355] Haque R, Mazumder DN, Samanta S, Ghosh N, Kalman D, Smith MM (2003). Arsenic in drinking water and skin lesions: dose-response data from West Bengal, India. Epidemiology.

[b21-ehp0114-000355] Hassan MM, Atkins PJ, Dunn CE (2005). Social implications of arsenic poisoning in Bangladesh. Soc Sci Med.

[b22-ehp0114-000355] Hoque BA, Mahmood AA, Quadiruzzaman M, Khan F, Ahmed SA, Shafique SA (2000). Recommendations for water supply in arsenic mitigation: a case study from Bangladesh. Public Health.

[b23-ehp0114-000355] Josephson J (2002). The slow poisoning of Bangladesh: metals in drinking water. Environ Health Perspect.

[b24-ehp0114-000355] Kurokawa M, Ogata K, Idemori M, Tsumori S, Miyaguni H, Inoue S (2001). Investigation of skin manifestations of arsenicism due to intake of arsenic-contaminated ground-water in residents of Samta, Jessore, Bangladesh. Arch Dermatol.

[b25-ehp0114-000355] Milton A, Rahman M (1999). Environment pollution and skin involvement pattern of chronic arsenicosis in Bangladesh. J Occup Health.

[b26-ehp0114-000355] Mukherjee SC, Rahman MM, Chowdhury UK, Sengupta MK, Lodh D, Chanda CR (2003). Neuropathy in arsenic toxicity from groundwater arsenic contamination in West Bengal, India. J Environ Sci Health A Tox Hazard Subst Environ Eng.

[b27-ehp0114-000355] Piechulek H, Al-Sabbir A, Mendoza-Aldana J (2003). Diarrhea and ARI in rural areas of Bangladesh. Southeast Asian J Trop Med Public Health.

[b28-ehp0114-000355] Rahman MM, Mukherjee D, Sengupta MK, Chowdhury UK, Lodh DC, Roy S (2002). Effectiveness and reliability of arsenic field testing kits: are the million dollar screening projects effective or not?. Environ Sci Technol.

[b29-ehp0114-000355] Rahman MT 2002. Perception and societal response to hazard mitigation: an example of arsenic contamination of drinking water in Bangladesh. Univ Texas Austin Undergrad Res J 1:44–52. Available: http://www.utexas.edu/research/student/urj/2002/Rahman.pdf [accessed 2 February 2005].

[b30-ehp0114-000355] Smith AH, Goycolea M, Haque R, Biggs ML (1998). Marked increase in bladder and lung cancer mortality in a region of northern Chile due to arsenic in drinking water. Am J Epidemiol.

[b31-ehp0114-000355] Smith AH, Lingas EO, Rahman M (2000). Contamination of drinking-water by arsenic in Bangladesh: a public health emergency. Bull WHO.

[b32-ehp0114-000355] Van Geen A, Ahsan H, Horneman AH, Dhar RK, Zheng Y, Hussain I (2002). Promotion of well-switching to mitigate the current arsenic crisis in Bangladesh. Bull WHO.

[b33-ehp0114-000355] Wasserman GA, Liu X, Parvez F, Ahsan H, Factor-Litvak P, van Geen A (2004). Water arsenic exposure and children’s intellectual function in Araihazar, Bangladesh. Environ Health Perspect.

[b34-ehp0114-000355] WHO 2001a. Arsenic Contamination in Groundwater Affecting Some Countries in the South-east Asia Region. WHO Regional Committee, Fifty-fourth Session, Provisional Agenda Item 9. Available: http://w3.whosea.org/rc54/pdf/rc54-8.pdf [accessed 1 February 2006].

[b35-ehp0114-000355] WHO 2001b. Arsenic in Drinking Water. Available: http://www.who.int/mediacentre/factsheets/fs210/en/ [accessed 1 February 2006].

[b36-ehp0114-000355] Yeh S (1973). Skin cancer in chronic arsenicism. Hum Pathol.

